# The early predictive value of routine laboratory tests on the severity of acute pancreatitis patients in pregnancy: a retrospective study

**DOI:** 10.1038/s41598-020-66921-x

**Published:** 2020-06-22

**Authors:** Di Jin, Jixue Tan, Jingsun Jiang, Dana Philips, Ling Liu

**Affiliations:** 1 Department of Gastroenterology and Hepatology, West China Hospital, Sichuan University, Chengdu, 610041 China; 2 West China School of Medicine, Sichuan University, Chengdu, 610041 China; 3 Queen Mary School, Medical College of Nanchang University, Nanchang, 330031 China

**Keywords:** Gastroenterology, Risk factors

## Abstract

Acute pancreatitis in pregnancy (APIP) varies in severity from a self-limiting mild condition to a severe life-threatening condition, and its severity is significantly correlated with higher risks of maternal and foetal death. This study evaluated the early predictive value of routine laboratory tests on the severity of APIP patients. We enrolled 100 patients with APIP in West China Hospital. Initial routine laboratory tests, including the biochemistry and hematologic tests were collected within 48 hours after the onset of APIP. For predicting SAP in AP, LDH had the highest specificity of 0.879. RDW was a suitable predictive marker as it had the sensitivity of 0.882. Lower levels of triglycerides (<4.72 mmol/L) predicted mild AP of APIP, with an area under the curve (AUC) of 0.724, and a negative predictive value of 0.80. Furthermore, a risk score was calculated based on white blood cells, neutrophils, RDW, LMR and LDH, as an independent marker (adjusted odds ratio = 3.013, 95% CI 1.893 to 4.797, P < 0.001), with the highest AUC of 0.906, a sensitivity of 0.875 and a specificity of 0.828. In conclusion, the risk score we recommended was the powerful marker to aid in the early prediction of the severity of APIP patients.

## Introduction

Acute pancreatitis (AP) is an acute inflammation of the pancreas that varies in severity from a self-limiting mild condition to a severe condition with multiple organ dysfunction syndrome or infected pancreatic necrosis^1^. According to the revision of the Atlanta classification in 2012^2^, AP is classified into mild acute pancreatitis (MAP), moderately severe acute pancreatitis (MSAP) and severe acute pancreatitis (SAP). Over the past decades, the mortality of SAP patients has reached 30%, despite many advanced management strategies, including fluid resuscitation, organ supportive care, early enteral nutrition and necessary endoscopic therapy^1^. AP in pregnancy (APIP) is a rare but severe condition with the incidence of approximately 1 out of 1,000-12,000 pregnant woman^3,4^. The maternal and perinatal mortality rates are still as high as 3.3% and 11.6–18.7%^5,6^, respectively.

The severity of APIP is significantly correlated with the higher risks of maternal and foetal death^6,7^. Early recognition of AP severity at emergency departments is critical so that prompt treatment can be provided for individual patients. Several scores, not specific for pregnant patients, are often used as a guide to evaluate the severity of APIP^8^. However, these scores may not relate to the APIP patients and the severity of critical diseases in pregnant patients^9,10^. Furthermore, they are recommended to be processed 72 hours after the onset of AP, which reduce the predictive value. It is necessary to identify convenient and effective laboratory markers for early prediction of the severity of APIP in clinical settings.

Routine laboratory tests, including routine biochemistry and haematology tests, are conveniently used in emergency centres. White blood cells and neutrophil counts are typically used markers for predicting the severity in non-pregnant AP patients^11^. Combined markers of the routine test are recommended to evaluate the severity of inflammatory diseases, including red cell distribution width (RDW)^12^, neutrophil–lymphocyte ratio (NLR)^13^, lymphocyte–monocyte ratio (LMR)^14^, platelet-lymphocyte ratio (PLR)^15^ and prognostic nutritional index (PNI)^16^. Among them, RDW^17,18^ and NLR^19^ have been reported to predict the severity of AP in non-pregnant patients. Recently, Ilhan *et al*.^20^ reported that NLR might be used as an early marker of APIP by studying 14 APIP patients compared to normal controls.

In this study, we retrospectively reviewed 100 cases of APIP and classified the patients into mild, moderate and severe groups. We evaluated the early predictive value of routine laboratory tests on the severity of APIP patients. Routine laboratory tests within 48 hours after the APIP onset were collected to evaluate the predictive values of these tests on the severity of APIP.

## Results

### Patient characteristics

A total of 100 patients with APIP were enrolled in the study, including 40 MAP, 26 MSAP and 34 SAP, respectively. Six patients were excluded from the analysis, including those having chronic cardiopulmonary disease (n = 4) and those arriving at the hospital 48 hours after the APIP onset (n = 2). Table 1 includes the baseline characteristics of the patients. There were no significant differences in age (*P* = 0.475) and gestational weeks (*P* = 0.621) among the three groups (MAP, MSAP and SAP). In terms of aetiology, there was significant difference of aetiology between the MAP and SAP group (P = 0.010). SAP patients had higher percent of hypertriglyceridemia as compared to MAP patients (55.9%, 19/34 vs 22.5%, 9/40, *P* = 0.003). The median gestational weeks of MAP, MSAP and SAP were 30.5, 33.5 and 31.0 weeks, respectively (*P* > 0.05).

**Table 1:** Clinical characteristics and laboratory findings in patients with acute pancreatitis in pregnancy.

Variables	MAP(n = 40)	MSAP(n = 26)	SAP(n = 34)	*P* Value			
				All groups	1 vs 2	2 vs 3	1 vs 3
Age (years)	28.40 ± 6.14	29.81 ± 6.01	28.06 ± 4.96	0.475	0.995	0.732	1.000
Pregnancy weeks	30.5(11–38)	33.5(13–37)	31.0(18–40)	0.621	—	—	—
Etiology(a/b/c)	4/9/27	3/12/11	3/19/12	0.035	0.099	0.745	0.010
Hospital stay(days)	7.5(1–26)	8.0(1–30)	16.0(6–53)	<0.001	1.000	<0.001	<0.001
Fetal death (n)	0	0	1	—	—	—	—
Maternal death (n)	3	3	10	—	—	—	—
WCC(×10^9/L)	12.37 ± 4.52	14.10 ± 3.60	16.82 ± 5.08	<0.001	0.396	0.067	<0.001
Neu(×10^9/L)	10.80 ± 4.51	12.73 ± 3.52	15.18 ± 4.84	<0.001	0.254	0.106	<0.001
Glucose(mmol/L)	5.12(3.44–40)	6.57(3.53–17.9)	8.49(3.71–39.5)	0.004	0.183	0.275	0.004
Albumin(g/L)	35.89 ± 4.37	34.70 ± 4.41	32.62 ± 5.50	0.016	0.974	0.300	0.013
RDW(%)	14.75 ± 1.85	15.45 ± 1.66	15.77 ± 1.61	0.039	0.335	1.000	0.040
NLR	10.39(2.25–47.6)	16.77(4.67–44.62)	16.70(4.70–47.5)	0.002	0.052	1.000	0.002
LMR	1.93(0.71–6.5)	1.61(0.53–15.75)	1.24(0.38–10)	0.002	1.000	***0.025***	0.003
PNI	40.81 ± 4.87	39.02 ± 5.32	37.07 ± 5.97	0.014	0.568	0.506	0.011
BUN(mmol/L)	3.06(1.21–6.11)	3.17(0.94–10.44)	3.77(1.51–28.91)	0.035	1.000	0.203	0.039
Scr(umol/L)	45(27.6–67)	48(30–112)	57(19–732.1)	0.003	0.120	0.128	0.001
ALT(IU/L)	26(6–355)	18(8–128)	17(4–107)	0.028	0.299	1.000	0.028
LDH(IU/L)	170(114–303)	207(110–938)	288(144–1146)	<0.001	*0.340*	***0.032***	<0.001
Calcium(mmol/L)	2.20(1.72–2.47)	2.14(1.70–2.46)	2.00(0.99–2.41)	0.031	1.000	0.109	0.047
TG(mmol/L)	3.35(0.89–22.93)	13.5(2.22–27.77)	14.12(1.54–85.46)	0.001	***0.029***	1.000	0.002
TC(mmol/L)	5.15(3–39.24)	9.86(3.46–24.01)	13.5(2.89–36.14)	0.011	0.168	1.000	0.010

Continuous variables are presented as mean ± SD or median (range).

Etiology (a/b/c), a, b and c represent gallstone, hyper triglyceridaemia and other etiologies, respectively.

1 vs 2, MAP group versus MSAP group; 2 vs 3, MSAP group versus SAP group; 1 vs 3, MAP group versus SAP group.

Compared to the MAP group, the significantly changed markers in the SAP group are shown in Table 1. Compared to the MAP group, white blood cell count (WCC), neutrophil count (Neu), glucose, albumin, RDW, NLR, blood urea nitrogen (BUN), serum creatinine (Scr), aminotransferase (ALT), lactate dehydrogenase (LDH), triglyceride (TG) and total cholesterol (TC) increased significantly (*P* < 0.05) in the SAP group, whereas LMR, PNI and calcium decreased significantly (*P* < 0.05). The other results, including amylase, lipase, coagulation function and creatine kinase had no significant difference among the three groups (data not shown). TG was the only significantly different marker between MAP (3.35 mmol/L) and MSAP (13.5 mmol/L) group (*P* = 0.029). Significantly lower LMR (*P* = 0.025) and higher LDH (*P* = 0.032) were observed in the SAP group compared with the MSAP group.

### The markers’ power for predicting SAP

**Figure 1:**
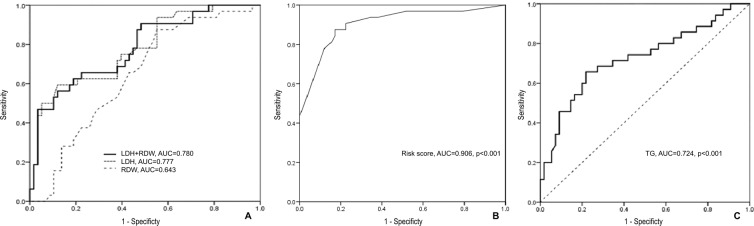
(**A**) ROC curves analysis for predicting SAP by LDH and RDW. The area under curve AUC for combination curve of RDW and LDH was 0.780 (95%CI 0.680 to 0.880. P < 0 .001). The Z test showed there were no significant differences in AUC between the combination group and the single LDH group (P > 0.05). (**B**) ROC curves analysis for predicting SAP by risk score. The AUC of the risk score was 0.906, with a sensitivity of 0.875 and a specificity of 0.828. (**C**) ROC curves analysis for predicting MAP by TG. The AUC for TG was 0.724 (P < 0.001, 95% CI 0.612 to 0.837, cutoff value = 4.720). AUC, area under curve; ROC, receiver operating characteristic curve; AUC, area under curve; SAP, severe acute pancreatitis; LDH, lactate dehydrogenase; RDW, red cell distribution width; MAP, mild acute pancreatitis; TG, triglyceride.

ROC curves were constructed to compare the values of the changed markers to predict the severity in APIP patients and find cut-off values for further logistic regression. AUC and the optimal cut-off values are shown in Table 2. The ability of LDH to predict SAP was highest (AUC = 0.777, *P* < 0.001, 95% confidence interval (CI) 0.676 to 0.877). LMR (AUC = 0.715, *P* < 0.001), WCC (AUC = 0.713, *P* = 0.001) and Neu (AUC = 0.707, *P* = 0.001) had moderate level of accuracy to predict. RDW had the highest sensitivity (0.882) with the lowest negative likelihood ratio (0.27). LDH had the highest specificity (0.879) with the highest positive likelihood ratio (4.91). Therefore, we selected RDW and LDH for combination. The AUC for combination curve of RDW and LDH was 0.780 (95% CI 0.680 to 0.880. *P* < 0 .001) (Fig. 1A). The Z test showed that there were no significant differences in AUC between the combination group and the single LDH group (*P* > 0.05). Furthermore, the AUC of TG for prediction of MAP was 0.724 (*P* < 0.001, 95% CI 0.612 to 0.837, cut-off value = 4.720) (Fig. 1C).

**Table 2:** Biochemical indexes for predicting SAP in the patients with APIP.

Variables	AUC	95%CI	*P* Value	Cut-off	Sensitivity	Specificity	+LR	-LR
WCC(×10^9/L)	0.713	0.607 to 0.818	0.001	12.99	0.765	0.576	1.80	0.41
Neu(×10^9/L)	0.707	0.602 to 0.813	0.001	11.64	0.794	0.561	1.81	0.37
Glucose(mmol/L)	0.666	0.543 to 0.788	0.007	6.62	0.706	0.636	1.94	0.46
Albumin(g/L)	0.659	0.547 to 0.771	0.009	35.65	0.515	0.824	2.93	0.59
RDW(%)	0.656	0.547 to 0.764	0.011	14.35	0.882	0.439	1.57	0.27
NLR	0.659	0.550 to 0.769	0.009	12.79	0.824	0.470	1.55	0.37
LMR	0.715	0.605 to 0.824	<0.001	1.51	0.727	0.735	2.74	0.37
PNI	0.657	0.543 to 0.771	0.010	38.60	0.652	0.735	2.46	0.47
BUN(mmol/L)	0.659	0.538 to 0.779	0.011	4.10	0.455	0.877	3.70	0.62
Scr(umol/L)	0.688	0.567 to 0.809	0.002	48.70	0.758	0.600	1.90	0.40
ALT(IU/L)	0.630	0.517 to 0.744	0.036	25.00	0.818	0.415	1.40	0.44
ALT/AST	0.698	0.576 to 0.744	0.001	0.61	0.636	0.800	3.18	0.46
LDH(IU/L)	0.777	0.676 to 0.877	<0.001	263.00	0.594	0.879	4.91	0.46
Calcium(mmol/L)	0.668	0.549 to 0.787	0.009	2.075	64.5	65.6	1.38	0.28
TG(mmol/L)	0.661	0.543 to 0.779	0.012	5.16	0.781	0.552	1.74	0.40
TC(mmol/L)	0.649	0.525 to 0.772	0.020	7.03	0.750	0.569	1.74	0.44

−LR, negative likelihood ratio; +LR, positive likelihood ratio; AUC, area under the receiver operating characteristic curve; PNI, prognostic nutritional index, were calculated as albumin (g/L) plus 5 × total lymphocyte count (10^9/L).

Making a brief conclusion, for predicting SAP in AP, LDH had the highest AUC, with a sensitivity of 0.594 and a specificity of 0.879. RDW was a suitable predictive marker as it had the highest sensitivity (0.882) and lowest negative likelihood ratio (0.27).

### The relationship between markers to SAP

The binary logistic regression models were used to find relationship between markers and SAP. Univariate analysis revealed that higher level of WCC, Neu, glucose, RDW, NLR, BUN, Scr, LDH, TG, TC and lower level of albumin, LMR, calcium were related to SAP (Table 3) in unadjusted model or adjusted for ages and pregnancy weeks. Adjusted for all variables except themselves, higher levels of RDW (>14.35%, adjusted odds ratio (OR) = 5.065, 95%CI 1.199 to 21.398, *P* = 0.027), LDH (>263 IU/L, adjusted OR = 23.568, 95% CI 4.809 to 115.500, *P* < 0.001) and lower LMR (<1.51, adjusted OR = 8.567, 95% CI 2.049 to 35.824, *P* = 0.003) were independent factors for predicting SAP in patients with AP during pregnancy by multivariate analysis (Table 4). Moreover, we calculated a risk score. After adjusting for ages and pregnancy weeks, the risk score was an independent factor to predict SAP in APIP (OR = 3.013, 95% CI 1.893 to 4.797, *P* < 0.001). The AUC of the risk score was 0.906, with the cut-off value as 3.963. It had a sensitivity of 0.875 and a specificity of 0.828 (Fig. 1B).

**Table 3:** Univariate analysis of factors for predicting SAP in patients with APIP.

Factors	OR	95%CI	*P* Value	OR	95%CI	*P* Value
	Model 1			Model 2		
WCC( > 12.99 vs <12.99, ×10^9/L)	4.714	1.758 to 12.639	0.002	4.874	1.800 to 13.198	0.002
Neu(>11.64 vs <11.64, ×10^9/L)	5.333	1.909 to 14.902	0.001	5.372	1.917 to 15.052	0.001
Glucose(>6.62 vs <6.62, mmol/L)	6.158	2.334 to 16.244	<0.001	7.146	2.576 to 19.820	<0.001
Albumin(<35.65 vs>35.65, g/L)	4.643	1.664 to 12.957	0.003	5.399	1.854 to 15.723	0.002
RDW( > 14.35 vs <14.35, %)	5.687	1.768 to 18.297	0.004	5.637	1.748 to 18.172	0.004
NLR( > 12.79 vs <12.79)	3.521	1.260 to 9.838	0.016	—	—	—
LMR( < 1.51 vs>1.51)	6.708	2.563 to 17.555	<0.001	7.240	2.700 to 19.411	<0.001
PNI( < 38.6 vs>38.6)	4.182	1.641 to 10.657	0.003	4.731	1.777 to 12.595	0.002
BUN( > 4.1 vs <4.1, mmol/L)	5.515	1.990 to 15.283	0.001	6.072	2.060 to 17.894	0.001
Scr(>48.7 vs <48.7, umol/L)	4.909	1.880 to 12.821	0.001	5.548	2.033 to 15.141	0.001
ALT/AST( < 0.612 vs>0.612)	10.648	3.692 to 30.709	<0.001	14.955	4.568 to 48.965	<0.001
LDH( > 263 vs <263, IU/L)	3.463	1.400 to 8.567	0.007	4.055	1.549 to 10.618	0.004
TG( > 5.12 vs <5.12, mmol/L)	3.444	1.329 to 8.925	0.011	3.964	1.454 to 10.787	0.007
TC( > 7.03 vs <7.03, mmol/L)	3.692	1.424 to 9.575	0.007	4.651	1.643 to 13.169	0.004

Model 1: unadjusted model.

Model 2: adjusted for ages and pregnancy weeks.

“—” means the *P* value of Omnibus tests of model coefficients > 0.05.

**Table 4:** Multivariate analysis of factors for predicting SAP in patients with APIP.

Factors	Coefficient	OR	95%CI	*P* Value
WCC(>12.99 vs <12.99, ×10^9/L)	−1.144	0.318	0.023 to 4.332	0.390
Neu(>11.64 vs <11.64, ×10^9/L)	2.534	12.599	0.785 to 202.151	0.074
RDW(>14.35 vs <14.35, %)	1.622	5.065	1.199 to 21.398	0.027
LMR(<1.51 vs>1.51)	2.148	8.567	2.049 to 35.824	0.003
LDH(>263 vs <263,, IU/L)	3.160	23.568	4.809 to 115.500	<0.001

Model 3: adjusted for all inputs except itself. Inputs: Neu, RDW, LMR, LDH, WCC.

## Discussion

Here, we report the predictive values of routine laboratory tests on the severity of APIP within 48 hours after the onset of APIP. Compared to the MAP group, several markers, including WCC, neutrophil count, glucose, albumin, RDW, NLR, blood urea nitrogen, serum creatinine, alanine aminotransferase, LDH, triglyceride and total cholesterol in the SAP group, increased significantly (*P* < 0.05), whereas LMR, PNI and calcium decreased significantly (*P* < 0.05). Increased levels of LDH, RDW > 14.35 and LMR < 1.51 were the independent factors that aided in the early prediction of the severity of APIP patients. In order to find a more effective predicator, the risk score was calculated based on WCC, neutrophils, RDW, LMR and LDH (Table 4). As an independent index, the risk score had the highest AUC of 0.906, with a sensitivity of 0.875 and a specificity of 0.828. Therefore, the risk score was recommended as the most powerful marker to aid in the early prediction of the severity of APIP patients in the study.

APIP is a rare type of acute pancreatitis. Due to the lack of sufficient cases for severity grading, most of the previous studies only retrospectively reported the clinical features of APIP, including the incidence, causes, clinical characteristics and outcomes of patients^3,5,9,21^. Zhang *et al*. identified a panel of blood tests to predict APIP based on comparing 59 APIP patients with 179 normal pregnant women^22^. Ilhan, M. reported that NLR might be used as an early marker of AP, dependent on 14 APIP patients without the grading of severity^20^. In 2018, Luo *et al*. reviewed clinical manifestation of 121 APIP, the largest sample size to date, and divided APIP patients into MAP, MSAP and SAP groups. Although several indicators for the severity of the APIP were studied^6^, their early prediction values were not studied. Also, the proportion of SAP in their study was 13.22% (16/121), significantly lower than that in our study (36%, 36/100).

LDH is a cytoplasm enzyme that participates in the conversion of lactate to pyruvic acid and back. It can be found extensively in all tissues, at higher concentration in the heart, kidney and skeletal muscles. Elevated LDH is observed in tissue injury, necrosis, hypoxia or malignancies. LDH isoenzymes (LDH-4) were reported to be more valuable than Ranson’s criteria in the early assessment of the severity of acute pancreatitis, according to the 1992 Atlanta criteria of acute pancreatitis^23^. However, the predominant LDH isoenzymes are LDH-2 and LDH-3, which implies that the damage to the pancreas as well as to the heart, lung or renal contributes to the higher value of serum LDH in AP. This is supported by the recent study, reported by Cui *et al*.^24^, that serum LDH on admission is independently associated with persistent organ failure in AP. LDH is also recommended to be included in the decision tree model to predict SAP^25^. However, no study has been reported the relationship of LDH to the severity of APIP patients. In our study, we report that significantly increased LDH had the highest AUC (0.777), with a cut-off value if 263 IU/L and a specificity of 0.879, which can be treated as one of the best predictors for SAP in APIP patient in our study. Although inter-laboratorial differences of LDH analysis exists, our results support that higher levels of LDH > 263 IU/L, 19.5% higher than the normal high range (normal range 110 – 220 IU/L), was an independent factor associated of the severity of APIP.

RDW is a widely-used marker for the quantification of the extent of erythrocyte anisocytosis. It is calculated by dividing standard deviation of red blood cell volume by mean corpuscular volume (MCV) and multiplying by 100 to express the results as percentages, routinely performed as part of a complete blood count^26^. Several studies have reported that RDW, as an independent marker, is associated with the severity of acute pancreatitis^17,18^. RDW > 13.55% was predicted to be related to SAP at early admission stage^17^. In our study, we reported that the normal level of RDW was a suitable marker to exclude SAP, determined by the highest sensitivity (88.2%) in our study. After adjusting for all inputs except itself, higher RDW than that in non-pregnant AP patients was an independent factor of SAP ( > 14.35%, adjusted OR = 5.065, 95% CI 1.199 to 21.398, *P* = 0.027) (Table 4).

The systemic inflammatory response plays a crucial role in the pathogenesis and progression of SAP^1^. Several systemic inflammatory indicators related to leukocytes, such as WCC, Neu, LMR and NLR have been explored to predict the prognosis in a wide variety of diseases^13,14^. In our study, we reported that the AUC of WCC, Neu, and LMR in APIP were greater than 0.7, the first two in accordance with previous study^11^, but multivariate logistic regression models found that only LMR was an independent factor related to the severity of APIP. Lymphocytes are considered to play crucial roles in the anti-inflammatory reaction, whereas the degree of monocyte activation has been recognised as one of the most important factors that determine the severity of AP^27^. Monocytes produce various cytokines and inflammatory mediators, including tumour necrosis factor α (TNFα), interleukin (IL)−1β, IL-6, monocyte chemoattractant protein (MCP)−1 or CCL2 and platelet-activating factor^28^. Depleting monocytes^29^ or pre-treatment with macrophage migration inhibitory factor antibodies^30^ significantly improved the survival in animal models of AP. In our study, we first reported that the ratio of lymphocytes to monocytes counts was significantly decreased in the SAP group of APIP patients. Lower LMR was an independent factor of SAP in pregnancy, with a cut-off value of 1.51 (adjusted OR = 8.567, 95% CI 2.049 to 35.824, *P* = 0.003) (Table 4).

We also observed that triglyceridaemia is the only significantly different marker between the MAP to the MSAP or SAP patients. Most pregnant women have a modest increase in serum TG during the third trimester due to a direct effect of oestrogen on liver lipoprotein synthesis and from decreased clearance of tri-glycerides due to hormone suppression of lipoprotein lipase activity in the liver and adipose tissue^31^. Additionally, the fat necrosis in parenchymal and peripancreatic adipose tissue can release fatty acid into the blood and participate in the increasing TG levels^32^. Our study supports that lower TG levels (<4.72 mmol/L) within 48 hours after APIP onset may be treated as the predictor of mild prognosis for APIP patients, with an AUC of 0.724 and a negative predictive value of 0.80.

An elevated serum amylase and/or lipase level higher than three times normal should be taken into account during pregnancy, as in non-pregnant women, for the diagnosis of AP^33^. However, neither amylase nor lipase had predictive value on the severity of APIP, since there was no significant difference of amylase and lipase among MAP, MSAP and SAP patients in our study (amylase (U/L): 371(47–3420), 250(21–2854), 467(68–2883), respectively, *P* = 0.227; lipase(U/L): 514.5(19–2090), 371(24–2763), 717.5(79–7099), *P* = 0.166, respectively). BUN, calcium and neutrophils are traditionally associated with severe acute pancreatitis. However, these variables were not independent factors associated with the severity of SAP in our study.

An elevated serum WCC, neutrophils, RDW and lower level of LDH have been reported in pregnant women, as compared to the normal population^20,22,34,35^. The elevated trend of WCC and neutrophil was also shown in previous study as compared APIP patients with pregnant women^20^. APIP patients with pathological condition are enrolled in our hospital, a national centre for critical diseases, whereas the normal pregnant women are not cared in our centre. Therefore, we had no chance to compare the APIP patients to the normal pregnant women in the study. However, through clinical manifestations, such as typical abdominal pain or ultrasonography manifestation, it is not difficult to distinguish APIP from normal pregnant women. To our knowledge, no studies have reported the difference of LDH, RDW and LMR between pregnancy and APIP patients and our study firstly reports the risk score based on these markers to aid early prediction on the severity of APIP patients.

Our study has several limitations. Firstly, potential bias exists in this retrospective single-centre study at a referral academic hospital, in which the patients may represent an overall higher risk of SAP, with the percentage of 36% in our study. We only enrolled the patients with complete medical records of APIP, those patients who were not admitted to the hospital were excluded from the analyses, although the number was only 2. Therefore, a larger, multi-centre prospective study is needed to validate the results. Secondly, not all patients tested arterial gas blood gas analysis in the medical record to evaluate the APACHE scores or Ransons scores. Only 17 patients (6 in MAP group, 1 in MSAP group, 10 in SAP group) reported CRP value in the retrospective study, based on medical records. The significant value of these scores and CRP value in APIP may be clarified in future prospective research. Thirdly, we only described the association of each of the predictors with the severity of AP, the underlying mechanisms that these factors are protective or dangerous in a pathophysiological sense need to be studied. Fourthly, other prediction models, such as using machine learning should be also constructed and confirmed in future research.

In conclusion, increased level of LDH, RDW > 14.35, LMR < 1.51 and the risk score were the independent factors that aided early prediction of the severity of APIP. Risk score was the most powerful marker in this retrospective study to evaluate SAP in APIP patients, with a sensitivity of 0.875 and a specificity of 0.828. Normal TG levels predict the mild prognosis of APIP. We believe that this data offers the possibility to use routine laboratory tests for early prediction of the severity of APIP, which will be helpful for the determination of therapeutic management and improvement of the outcomes.

## Methods

### Patients

This retrospective study enrolled patients with APIP who were hospitalised in West China Hospital in Chengdu, China between January 1, 2013 and December 31, 2016. The standard of diagnosis of AP includes at least two of three features^2^: 1) Prolonged and intense upper abdominal pain with nausea or vomiting; 2) threefold elevation of serum amylase and/or lipase levels above the normal range and 3) characteristic findings of AP on abdominal ultrasonography and/or computed tomography (CT) scan. The classification of acute pancreatitis was according to the revision of the Atlanta classification in 2012 ^2^. MAP was determined by absence of organ failure or local complications, Ranson <3 or APACHE II < 8. MSAP was determined by local complications and/or transient organ failure <48 h, Ranson ≥3, APACHE II ≥ 8. SAP were determined by persistent single or multiple organ failure >48 h, or Marshall score ≥2^36^. Acute gallstone pancreatitis was diagnosed by radiological evidence of abdominal ultrasonography^37^. Hypertriglyceridemic pancreatitis was diagnosed based on the Chinese guidelines for the management of acute pancreatitis (Shanghai, 2013) with a serum triglyceride ≥11.3 mmol/L^38,39^. Other aetiologies include dietary surfeit, medication and idiopathic factors after excluding gallstone, alcohol, hypertriglyceridemia, trauma, autoimmune and surgical factors^37,40^. The following patients were excluded: concomitant diseases (chronic vital organ failure and autoimmune diseases), regular medicinal treatments with non-steroidal anti-inflammatory drugs or immune suppression, arriving at the hospital more than 48 hours after the onset of APIP. The project was approved by Human Research Ethics Committee of West China Hospital, Sichuan University of China on June 13, 2018 and still has approval (reference number 2018–189). As this is a retrospective study, it was not appropriate or possible to involve patients or the public in the design, conduct, reporting or dissemination of our research. West China Hospital of Sichuan University, located in Chengdu, the provincial capital of Sichuan Province, is a national centre for the diagnosis and treatment of critical diseases in Western China. It covers the population around 83 million in Sichuan Province and 267 million in other 11 provinces, autonomous regions and municipalities, with the annual birth rate around 10‰. This large population ensures sufficient numbers of APIP cases for analysis in the study.

### Demographic information and laboratory tests

Demographic information, including ages, gestational weeks and aetiology, was collected from the medical records. Initial tests, including biochemistry and haematologic tests before treatment, were collected within 48 hours after the onset of APIP. A MODULAR P800 chemistry analyser (Roche Diagnostics, Indianapolis, Indiana, USA) and Roche reagents (Roche Diagnostics, Indianapolis, Indiana, USA) were used in the laboratory. The complete medical history was collected for accurate diagnosis and classification. All clinical data were retrieved from medical records and were collected separately by two people. The inconsistent data were adjusted by two researchers and resolved by agreement. NLR, LMR, PLR, NMR were ratios of two types of blood contents. PNI was calculated as albumin (g/L) plus 5 × total lymphocyte count (10^9/L)^16^. Risk score = (−1.144*white blood cell count status) + (2.534* neutrophil count status) + (1.622*RDW status) + (2.148*LMR status) + (3.160*LDH status).

### Statistical analysis

Variables are expressed as mean ± SD or median (range), as appropriate. The distribution of data was assessed using the Kolmogorov-Smirnov test. Multiple comparisons were performed by one-way analysis of variance or Kruskal-Wallis tests, as appropriate. The Levene test was used for testing the homogeneity of variance, and the Bonferroni method was used to adjust for multiple comparisons. Differences between two groups were assessed using the least significant difference method adjusted by the Bonferroni method or Dunnett-t test, as appropriate. Categorical variables were reported as number (frequency) and analysed by the Fisher exact test. The accuracy of each marker to predict SAP in AP was assessed using the receiver operating characteristic (ROC) curves. The *P* value was compared with the area under curves (AUC) with 0.5, and sensitivity, specificity, positive likelihood ratio (+LR) and negative likelihood ratio (−LR) were calculated for assessing diagnostic value. Multivariate logistic regression analyses were used to assess whether markers were independent factors for predicting SAP in patients with AP by unadjusted and adjusted models. A *P* value <0.05 was considered a statistically significant difference. Statistical analyses were conducted with SPSS V.24.0 (SPSS, Chicago, Illinois, USA).

### Ethics approval

The project was approved by Human Research Ethics Committee of West China Hospital, Sichuan university, China on 13 June 2018 and still has approval (reference number 2018-189).

### Patient consent

Obtained.
